# The first evidence for SLFN11 expression as an independent prognostic factor for patients with esophageal cancer after chemoradiotherapy

**DOI:** 10.1186/s12885-020-07574-x

**Published:** 2020-11-20

**Authors:** Takuma Kagami, Mihoko Yamade, Takahiro Suzuki, Takahiro Uotani, Shinya Tani, Yasushi Hamaya, Moriya Iwaizumi, Satoshi Osawa, Ken Sugimoto, Hiroaki Miyajima, Satoshi Baba, Haruhiko Sugimura, Junko Murai, Yves Pommier, Takahisa Furuta

**Affiliations:** 1 First Department of Medicine, Hamamatsu University School of Medicine, 1-20-1, Handayama, Higashi-ku, Hamamatsu, 431-3192 Japan; 2 Department of Endoscopic and Photodynamic Medicine, Hamamatsu University School of Medicine, Hamamatsu, Japan; 3 Department of Clinical Laboratory Medicine, Hamamatsu University School of Medicine, Hamamatsu, Japan; 4 Department of Diagnostic Pathology, Hamamatsu University School of Medicine, Hamamatsu, Japan; 5 Department of Tumor Pathology, Hamamatsu University School of Medicine, Hamamatsu, Japan; 6 Institute of Advanced Biosciences, Keio University, Turuoka, Yamagata, Japan; 7 Developmental Therapeutics Branch and Laboratory of Molecular Pharmacology, Center for Cancer Research, National Cancer Institute, Bethesda, MD USA; 8 Center for Clinical Research, Hamamatsu University School of Medicine, Hamamatsu, Japan

**Keywords:** SLFN11, Esophageal cancer, Chemoradiotherapy, Biomarker, Nedaplatin, DNA damage

## Abstract

**Background:**

Schlafen 11 (SLFN11) was recently identified as a dominant determinant of sensitivity to DNA-targeting agents including platinum-based drugs. SLFN11 also reportedly enhances cellular radiosensitivity. In this study, we examined the prognostic value of SLFN11 expression in esophageal squamous cell carcinoma (ESCC) patients treated with definitive chemoradiotherapy (dCRT), including the platinum derivative nedaplatin.

**Methods:**

Seventy-three patients with ESCC who received dCRT were examined. SLFN11 expression was analyzed in pre-dCRT biopsies using immunohistochemistry and evaluated using a histo-score (H-score). Correlation between the H-score and overall survival was analyzed. An H-score ≥ 51 was provisionally defined as indicating high SLFN11 expression. Viability assays were performed using previously established isogenic human cell lines differentially expressing SLFN11 to test the usefulness of SLFN11 as marker of response to the dCRT regimen.

**Results:**

High SLFN11 expression was independently associated with better prognosis in ESCC patients (hazard ratio = 0.295, 95% CI = 0.143–0.605, *p* = 0.001 for multivariate analysis). Kaplan-Meier survival curves showed that the prognostic value of high SLFN11 expression was most evident in patients at clinical stages II and III (*p* = 0.004). In in vitro study, SLFN11-proficient cells were highly sensitive to platinum derivatives compared to SLFN11-deficient cells.

**Conclusion:**

SLFN11 expression is an independent prognostic factor for ESCC patients treated with dCRT and a potential biomarker for treatment selection of ESCC. Examination of SLFN11 may be particularly useful for clinical Stage II–III patients who wish to choose dCRT (instead of surgery) to preserve esophageal function.

**Supplementary Information:**

The online version contains supplementary material available at 10.1186/s12885-020-07574-x.

## Background

Esophageal cancer is among the solid tumors with poor prognosis. There are two main pathological types: esophageal squamous cell carcinoma (ESCC) and esophageal adenocarcinoma. Treatment comprises endoscopic submucosal dissection, surgery, chemotherapy, radiotherapy or combinations of these. The type of treatment selected is usually determined by clinical stage, performance status, tolerance to anesthesia, and/or the wishes of each patient. Following reports by Herskovic et al. and Cooper et al. [1, 2], clinical trials of definitive chemoradiation therapy (dCRT), which combines platinum-based drug, 5-fluorouracil and irradiation, were initiated in unresectable esophageal cancer patients with/without distant lymph node metastasis (cT4/cM1-lym) in Japan [3]. Since verifying the therapeutic effectiveness and manageable tolerability of dCRT in these patients (median survival time, 9–13.6 months; 3-year survival rate, 23–30%) [3–6], dCRT has become a standard treatment for patients with clinical stage IVA or clinical stage IVB (cM1-lym) esophageal cancer [7]. Subsequent clinical trials have since been performed in Japanese patients at a resectable clinical stage [8–12]. Currently, dCRT is most often performed in patients at a resectable clinical stage (i.e. clinical stage I (cT1b), II and III) because clinical stage II–III patients tend to select dCRT (instead of surgery) to preserve esophageal function and quality of life. Importantly, Kato et al. [11] reported that the overall survival of clinical stage II and III ESCC patients who undergo surgery is similar to that of patients treated with dCRT, although the 3-year survival of dCRT-treated patients is 44.7%. Therefore, although the effectiveness of dCRT is comparable to that of surgery, the moderate survival rate implies that some clinical stage II and III patients are poor responders to dCRT. Currently, however, there is no way to predict responders or non-responders to dCRT in advance. The identification of predictive biomarkers is an unmet need.

The *Schlafen* (*SLFN*) family of genes, first identified in mice and since shown to be present only in mammals, is involved in multiple cellular processes, including growth and immune regulation and cellular differentiation [13, 14]. SLFN11, one of the 5 human SLFNs, is a putative DNA/RNA helicase concentrated in the nucleus [14] and has a dominant role in sensitizing malignant cells to DNA-targeting anti-cancer agents [13, 15]. SLFN11 selectively augments the anti-cancer effects of anti-cancer agents that target DNA replication, such as topoisomerase (TOP) inhibitors (TOP1 inhibitors: camptothecin, topotecan and irinotecan; TOP2 inhibitors: etoposide, mitoxantrone and doxorubicin), alkylating agents (cisplatin and carboplatin) and DNA synthesis inhibitors (gemcitabine and cytarabine) [15, 16]. High SLFN11 expression is also associated with hypersensitivity to poly (ADP-ribose) polymerase (PARP) inhibitors [17–19]. A common mechanism of action among these drugs is induction of DNA damage that causes replication fork stalling with cell cycle checkpoint activation, which is known as replication stress. Recently, we revealed that SLFN11 is recruited to replication forks under replication stress, where it persistently blocks replication. This explains why SLFN11-positive cells are selectively killed by replication stress-inducing drugs [13, 20]. SLFN11 also reportedly enhances cellular radiosensitivity [21].

Based on this background, we investigated SLFN11 expression levels in ESCC, and examined the correlation between SLFN11 expression level and prognosis in ESCC patients treated with dCRT. We also conducted an in vitro study to investigate the effect of SLFN11 on the anticancer drug-sensitivity of human cell lines.

## Methods

### Study design and quality management

This study was conducted under a retrospective/observational design and is in accordance with the STROBE Statement and REMARK guidelines [22]. All authors contributing to this clinical research completed an e-learning program, *Good Clinical Practice Education and Training (eAPRIN)* prior to the start of the study. The study protocol was in advance approved by the ethics committee of Hamamatsu University School of Medicine, Hamamatsu, Japan (Approval no. E18–185).

### Subjects

**Fig. 1:**
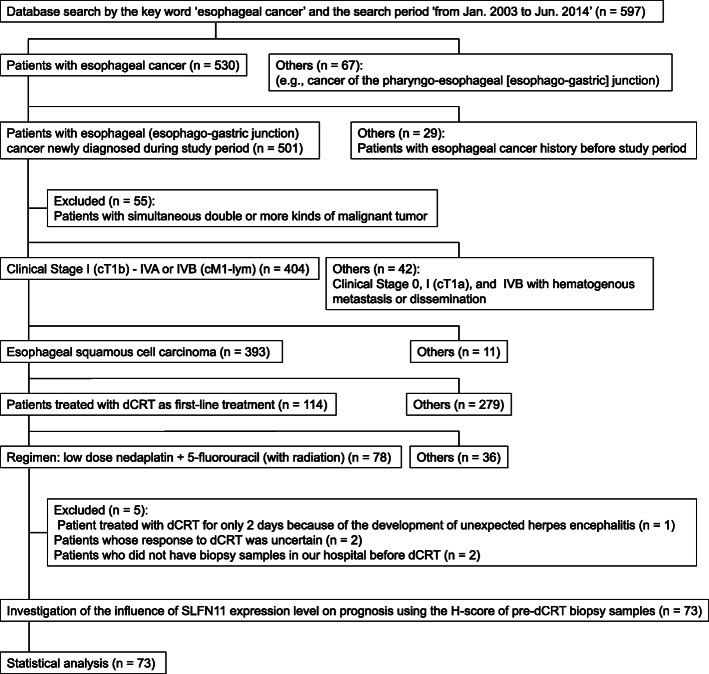
Participant flow. Abbreviations: cT1a, tumor invasion to the muscularis mucosa; cT1b, tumor invasion to the submucosa; cM1-lym, distant lymph node metastasis; dCRT, definitive chemoradiotherapy; H-score, histo-score

Our hospital database was searched using the expression “esophageal cancer” from January 2003 to June 2014. A total of 597 subjects were identified (Fig. 1). Among these, 78 ESCC patients received low-dose nedaplatin (cis-diammine-glycolatoplatinum) + 5-fluorouracil with concurrent radiation as first-line treatment and met the inclusion criteria (Table S1). Five of these patients were then excluded based on the exclusion criteria (Table S1), leaving 73 patients for analysis of the correlation between SLFN11 expression and overall survival.

### SLFN11 expression level and overall survival

**Fig. 2:**
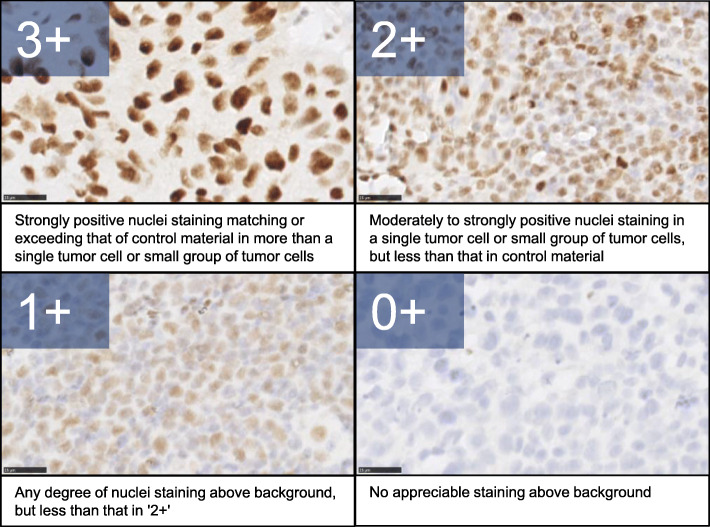
Semiquantitative scoring scheme for SLFN11 expression. SLFN11 level in the nucleus of tumor cells was determined using a 0+ to 3+ scale. Representative micrographs for each score are shown. The histo-score (H-score) for each patient was evaluated using the following formula: (% of cells 3+) × 3 + (% of cells 2+) × 2 + (% of cells 1+). H-score ≥ 51 was defined as high SLFN11 expression

Paraffin-embedded specimens obtained during esophagoscopy prior to dCRT were subjected to immunohistochemical staining with an anti-SLFN11 mouse monoclonal antibody (sc-515,071; Santa Cruz Biotechnology, Dallas, TX, USA) at 1:300 dilution [13] using previously described methods [23]. Two pathologists (S.B. and H.S.) evaluated the expression level of SLFN11 in each patient using a semi-quantitative method and calculated the histo-score (H-score) [24, 25] (Fig. 2). Patient medical records were accessed from the hospital information system. We analyzed the correlation between expression levels of SLFN11 and overall survival.

### Performance status

We evaluated the performance status of each patient in accordance with the Eastern Cooperative Oncology Group (ECOG) criteria [26]. Performance status of all subjects ranged from 0 to 2.

### Chemoradiotherapy

Dose intensities of nedaplatin and 5-fluorouracil were calculated as previously reported [27].

Each patient received a total of 60 Gy of radiation in principle. At least two courses of additional chemotherapy were then given after dCRT. We assessed therapeutic effect by computed tomography and esophagoscopy every 4–6 months after completion of dCRT.

### Radiation range and dosage

The standard dose of dCRT in western countries is 50.4 Gy/28 fractions [28]. In contrast, previous studies in Japan have commonly reported the use of 60 Gy/30 fractions [4, 6, 8, 10, 11, 29]. Our hospital uses 40 Gy/20 fractions to irradiate a field that includes the primary lesion, regional lymph nodes, and regions of distant lymph node metastasis when present. Additionally, 20 Gy/10 fractions were used to irradiate the primary lesion with a suitable margin.

### Cell lines and drugs

Human leukemia K562 and CCRF-CEM cell lines were grown in RPMI medium 1640 (1x, 11,875–093; Gibco, Thermo Fisher Scientific Inc., Waltham, MA, USA) which included 10% fetal bovine serum (100–106; Gemini Bio-Products Inc., West Sacramento, CA, USA) and 1% penicillin-streptomycin (15140–122, Gibco) at 37 °C in a 5% CO_2_ atmosphere. SLFN11-knockout cells were generated from CCRF-CEM and genetically modified K562 cell lines (K562 + vector and K562 + *SLFN11*) as described previously [18, 20]. Nedaplatin (143–09481), carboplatin (033–25,231) and 5-fluorouracil (068–01401) were obtained from Fujifilm Wako Pure Chemical Corp. (Osaka, Japan).

### Viability assay

Sensitivity to the drugs was measured by continuous exposure of cells to various concentrations of the drugs for 72 h. Wells in 384-well white plates (6,007,680; Perkin Elmer Life Sciences) were seeded with 2000 cells in 40 μl of medium per well. The ATPlite 1-step kit (PerkinElmer) was used to determine cellular viability, and luminescence was measured with an Infinite M200 (TECAN). ATP concentration in untreated cells was considered as 100%, and the viability (%) of treated cells was considered as ATP-treated cells/ATP-untreated cells × 100.

### Study size and statistical analysis

The ideal sample size was found to be at least 30 (15 subjects each with high and low SLFN11 expression) as recommended by a biostatistician (E.O.). Because SLFN11 has been reported to be inactivated in about 50% of cancer cell lines [20], we calculated that about half of the ESCC subjects would have high SLFN11, which in turn indicated the need for at least 30 eligible subjects (15/0.5) in order to enroll 15 ESCC subjects each with high and low SLFN11 expression. About 60% of ESCC patients in our hospital who received dCRT were provided the low-dose nedaplatin + 5-fluorouracil regimen. Finally, we determined that at least 50 patients were required (30/0.6).

Cox regression analysis was used to assess the association between clinicopathological variables and survival. The chi-squared test or Fisher’s exact test was used to analyze the relationship between clinicopathological variables and SLFN11. The survival of patients with high and low SLFN11 expression were compared using the Kaplan-Meier method and log-rank test. All analysis was conducted using SPSS ver. 24 (IBM, Madison Ave, NC, USA), and *p* values of less than 0.050 were considered to show statistical significance.

## Results

### Patients with high SLFN11 expression have longer overall survival

Subjects’ clinical demographic characteristics are presented in Table 1. Median (range) follow-up was 23 (12–44) months. The results of univariate and multivariate analyses of the effect of clinical parameters on overall survival are summarized in Table 2. Performance status, tumor size, clinical stage and high SLFN11 expression (H-score ≥ 51) were statistically associated with overall survival in univariate analyses (hazard ratio [HR] = 2.98, 95% confidential interval [CI] 1.43–6.19, *p* = 0.004; HR = 3.76, 95% CI 1.78–7.90, *p* <  0.001; HR = 5.12, 95% CI 2.45–10.95, p <  0.001; HR = 0.44, 95% CI 0.22–0.87, *p* = 0.018, respectively). In multivariate analysis, clinical stage and SLFN11 expression were independent prognostic factors (HR = 4.09, 95% CI 1.49–11.21, *p* = 0.006; HR = 0.295, 95% CI 0.14–0.61, *p* = 0.001, respectively) (Table 2).

**Table 1:** Characteristics of subjects with ESCC

Gender	Male	61 (83.6%)
	Female	12 (16.4%)
Age	Mean ± SD, (y)	69.0 ± 8.2
Weight	Mean ± SD, (kg)	53.9 ± 8.8
Height	Mean ± SD, (cm)	161.1 ± 8.0
Performance status	0	18 (24.7%)
	1	39 (53.4%)
	2	16 (21.9%)
	3	0 (0.0%)
eGFR	Mean ± SD, (ml/min/1.73 m2)	78.0 ± 20.5
Tumor location (primary site)	Ce	9 (12.3%)
	Ut	11 (15.1%)
	Mt	35 (47.9%)
	Lt	18 (24.7%)
	EGJ	0 (0.0%)
Number of pre-CRT biopsy samples	Median with range, (n)	2 (1–6)
Histological type	Well differentiated SCC	10 (13.7%)
	Moderately differentiated SCC	54 (74.0%)
	Poorly differentiated SCC	9 (12.3%)
	Basaloid SCC	0 (0.0%)
Depth of invasion	cTis	0 (0.0%)
	cT1a	0 (0.0%)
	cT1b	15 (20.5%)
	cT2	9 (12.3%)
	cT3	22 (30.1%)
	cT4a	12 (16.4%)
	cT4b	15 (20.5%)
Tumor size	Median with range, (cm)	5.0 (1.0–10.5)
Lymph node metastasis	cN0	24 (32.9%)
	cN1	12 (16.4%)
	cN2	32 (43.8%)
	cN3	5 (6.8%)
Distant metastasis	cM0	63 (86.3%)
	cM1-lym	10 (13.7%)
	cM1-hematogenous or (pleural/peritoneal) dissemination	0 (0.0%)
Clinical stage, TNM 8th	0	0 (0.0%)
	I (cT1b)	14 (19.2%)
	II	8 (11.0%)
	III	16 (21.9%)
	IVA	25 (34.2%)
	IVB (cM1-lym)	10 (13.7%)
	IVB with hematogenous metastasis or (pleural/peritoneal) dissemination	0 (0.0%)
SLFN11 expression	Mean ± SD, (H-score)	74.6 ± 77.7
Follow up periods	Median (range)	23 (12–44)

*Abbreviations*: *SD* standard deviation, *eGFR* estimated glomerular filtration rate, *CE* cervical esophagus, *Ut* upper thoracic esophagus, *Mt* middle thoracic esophagus, *Lt* lower thoracic esophagus, *EGJ* esophago-gastric junction, *cT1a* tumor invasion to the muscularis mucosa, *cT1b* tumor invasion to the submucosa, *cT2* tumor invasion to the muscularis propria, *cT3* tumor invasion to the adventitia, *cT4a* tumor invasion to the pleura, pericardium, azygos vein, diaphragm, or peritoneum, *cT4b* tumor invasion to other adjacent structures, such as the aorta, vertebral body, or trachea, *cN0* no regional lymph node metastasis, *cN1* metastasis in 1–2 regional lymph nodes, *cN2* metastasis in 3–6 regional lymph nodes, *cN3* metastasis in 7 or more regional lymph nodes, *cM0* no distant metastasis, *cM1-lym* distant lymph node metastasis, *CRT* chemoradiotherapy, *H-score* histo-score

All values indicate n (%) unless otherwise indicated

**Table 2:** Relationship between overall survival and clinicopathological variables

Variable		n	Univariate analysis		Multivariate analysis	
			HR (95% CI)	*P* value	HR (95% CI)	P value
Gender	Male	61	1 (reference)	0.657		
	Female	12	0.807 (0.313–2.080)			
Age (y)	< 65	26	1 (reference)	0.422		
	≥ 65	47	1.340 (0.656–2.738)			
Performance status	0 or 1	57	1 (reference)	0.004 ^a^	1 (reference)	0.066
	2	16	2.975 (1.431–6.186)		2.051 (0.954–4.410)	
Body surface area (m^2^)	< 1.50	30	1 (reference)	0.162		
	≥ 1.50	43	1.664 (0.815–3.398)			
eGFR (ml/min/1.73m^2^)	< 60	13	1 (reference)	0.838		
	≥ 60	60	1.096 (0.455–2.643)			
Tumor size (cm)	< 5	35	1 (reference)	< 0.001 ^a^	1 (reference)	0.332
	≥ 5	38	3.755 (1.784–7.903)		1.638 (0.605–4.436)	
Post-dCRT chemotherapy	–	17	1 (reference)	0.646		
	+	56	0.823 (0.358–1.891)			
Histological type	Differentiated	64	1 (reference)	0.876		
	Un-differentiated	9	1.079 (0.418–2.782)			
Clinical stage in UICC 8th edition	I (cT1b) - III	38	1 (reference)	< 0.001 ^a^	1 (reference)	0.006 ^a^
	IVA or IVB (cM1-lym)	35	5.177 (2.448–10.946)		4.085 (1.488–11.211)	
Radiation dose (Gy)	< 57	13	1 (reference)	0.670		
	≥ 57	60	1.229 (0.477–3.169)			
Nedaplatin dose intensity in dCRT (%)	< 90	45	1 (reference)	0.360		
	≥ 90	28	0.716 (0.351–1.463)			
5-fluorouracil dose intensity in dCRT (%)	< 90	47	1 (reference)	0.130		
	≥ 90	26	0.556 (0.261–1.188)			
SLFN11 expression	Low (< 51)	37	1 (reference)	0.018 ^a^	1 (reference)	0.001 ^a^
(H-score)	High (≥ 51)	36	0.438 (0.221–0.866)		0.295 (0.143–0.605)	

*Abbreviations*: *HR* hazard ratio, *CI* confidence interval, *eGFR* estimated glomerular filtration rate, *cM1-lym* distant lymph node metastasis, *dCRT* definitive chemoradiotherapy, *H-score* histo-score

^a^statistically significant

**Fig. 3:**
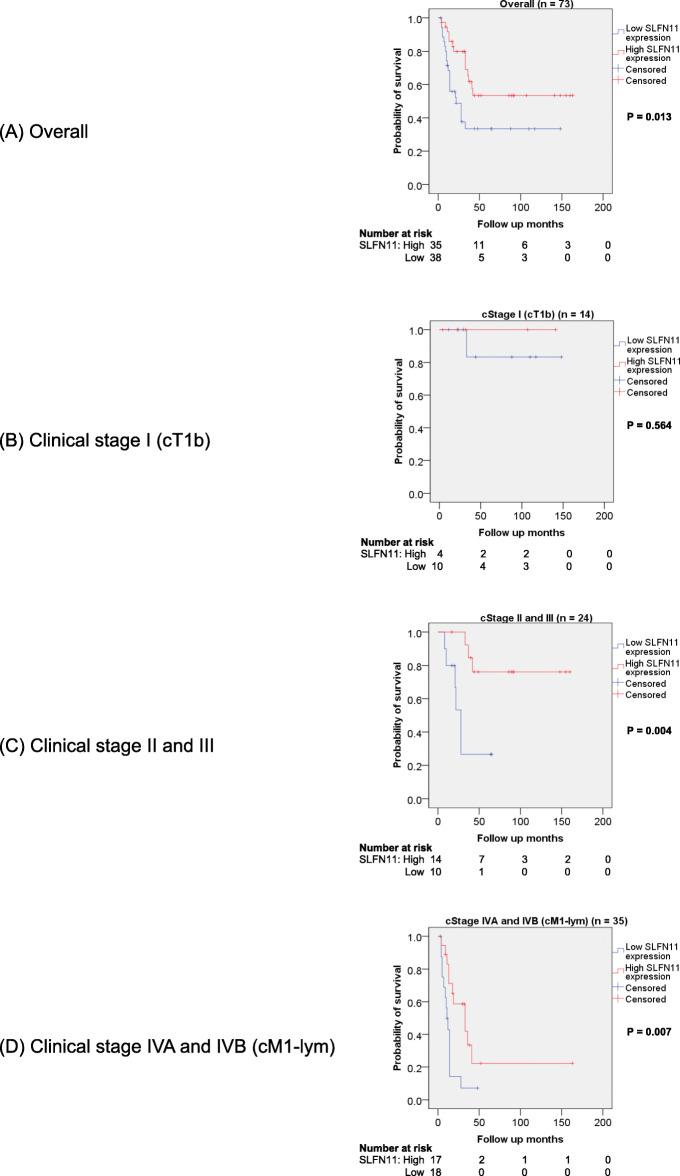
Kaplan-Meier curves of overall survival in ESCC patients in relation to SLFN11 expression. **a** In all subjects, prognosis after dCRT of the high SLFN11 group was significantly better than that of the low SLFN11 group (p = 0.013). **b** In clinical stage I (cT1b) patients, SLFN11-dependent differences in overall survival were not observed (p = 0.564). However, in clinical stage II + III (**c**) and clinical stage IVA + IVB (cM1-lym) patients (**d**), prognosis of the high SLFN11 group was better than that of the low SLFN11 group (p = 0.004 and p = 0.007, respectively). Abbreviations: ESCC, esophageal squamous cell carcinoma; dCRT, definitive chemoradiotherapy; clinical stage, clinical stage in UICC 8th edition; cT1b, tumor invasion to the submucosa; cM1-lym, distant lymph node metastasis

Kaplan-Meier survival curves showed that the overall survival of ESCC patients with high SLFN11 expression was significantly longer than that of patients with low SLFN11 expression (*p* = 0.013; Fig. 3a). When stratified by clinical stage, there was no statistically significant difference in overall survival between clinical stage I (cT1b) subjects with high and low SLFN11 expression, both of which had favorable overall survival rates (*p* = 0.564; Fig. 3b). In contrast, in clinical stage II + III and clinical stage IVA + IVB (cM1-lym), subjects with high SLFN11 expression had longer overall survival than those with low SLFN11 expression (*p* = 0.004, Fig. 3c; *p* = 0.007, Fig. 3d, respectively). Overall, these results suggest that high SLFN11 expression is associated with better prognosis in ESCC patients after dCRT.

### High SLFN11 expression is an independent factor for good prognosis

We analyzed the association between SLFN11 expression and clinicopathological variables (Table 3). Univariate analysis showed that no clinicopathological variable, including the number of pre-dCRT tumor biopsy specimens, met a significance level of 0.050. Age, however, met a significance level of 0.100 (*p* = 0.062), suggesting that there may be a significant association between SLFN11 expression and age in larger size studies. In our study, however, no clinicopathological variables affected the SLFN11 expression level. Therefore, SLFN11 expression is an independent factor associated with the overall survival of ESCC patients who received dCRT.

**Table 3:** Relationship between SLFN11 expression level and clinicopathological variables

Variable		SLFN-11 expression		*P* value
		Low	High	
		n (%)	n (%)	
Gender	Male	32 (52.5)	29 (47.5)	0.345
	Female	4 (33.3)	8 (66.7)	
Age	< 65	9 (34.6)	17 (65.4)	0.062
	≥ 65	27 (57.4)	20 (42.6)	
Number of pre-dCRT biopsy specimens including tumor tissue	1	5 (45.5)	6 (54.5)	0.781
	≥ 2	31 (50.0)	31 (50.0)	
Histological type	Differentiated	33 (51.6)	31 (48.4)	0.725
	Un-differentiated	3 (33.3)	6 (66.7)	
Depth of invasion	cT1b or T2	12 (50.0)	12 (50.0)	0.935
	cT3 - T4b	24 (49.0)	25 (51.0)	
Tumor size	< 5 cm	17 (48.6)	18 (51.4)	0.903
	≥ 5 cm	19 (50.0)	19 (50.0)	
Lymphatic metastasis	cN0	11 (45.8)	13 (54.2)	0.677
(regional lymph node)	cN1 - N3	25 (51.0)	24 (49.0)	
Distant metastasis	cM0	32 (50.8)	31 (49.2)	0.736
(distant lymph node)	cM1 -lym	4 (40.0)	6 (60.0)	

*Abbreviations*: *CI* confidence interval, *cT1b* tumor invasion to the submucosa, *cT2* tumor invasion to the muscularis propria, *cT3* tumor invasion to the adventitia, *cT4b* tumor invades adjacent structures, such as the aorta, vertebral body, or trachea, *cN0* no regional lymph node metastasis, *cN1* metastasis in 1–2 regional lymph nodes, *cN3* metastasis in 7 or more regional lymph nodes, *cM0* no distant metastasis, *cM1-lym* distant lymph node metastasis, *dCRT* definitive chemoradiotherapy

### Nedaplatin but not 5-fluorouracil causes SLFN11-dependent toxicity

To experimentally test the usefulness of SLFN11 as a marker of response to the dCRT regimen, we performed viability assays using previously established isogenic human cell lines differentially expressing SLFN11 (human leukemia CCRF-CEM SLFN11-proficient [parent] and -deficient [SLFN11-KO], and K562 SLFN11-deficient [K562 + vector] and -proficient [K562 + SLFN11] cell lines) [18] (Figure S1). Consistent with previous reports based on cancer cell database correlations [30, 31], SLFN11-proficient cells were highly sensitive to carboplatin and nedaplatin compared to SLFN11-deficient cells. In contrast, SLFN11 expression level did not affect sensitivity to 5-fluorouracil. Therefore, the SLFN11-dependent improvement in clinical outcome may be derived from the improved response of tumors expressing high SLFN11 to nedaplatin but not 5-fluorouracil.

## Discussion

Our study shows that high SLFN11 expression is an independent factor for good prognosis in ESCC patients treated with dCRT. Our data suggest that examination of SLFN11 level in biopsy samples may be useful for treatment selection (surgery or dCRT), and that further investigation of SLFN11 as a biomarker of treatment response in clinical settings is warranted.

### SLFN11 level and ESCC patient prognosis after dCRT

We observed a significant correlation between SLFN11 expression level in biopsy tissue before treatment and overall survival post-dCRT in ESCC patients. While dCRT combines three treatments, nedaplatin, 5-fluorouracil and irradiation, our study (Figure S1) and a report by Mu et al. [21] indicate that the improved outcome in tumors expressing high levels of SLFN11 is derived from the cells’ response to nedaplatin and irradiation but not 5-fluorouracil.

### SLFN11 expression and age

Although we found no significant correlation between SLFN11 expression and other clinicopathological variables (Table 3), SLFN11 expression levels tended to be lower in older patients. A previous report suggested that high levels of methylation of *SLFN11* were significantly associated with older age [32]. Bioinformatics analysis using CellMinerCDB (https://discover.nci.nih.gov/cellminercdb/) demonstrated a strong inverse correlation between *SLFN11* methylation in promoter regions and its mRNA expression level (r = − 0.750, *p* = 0.00013; Figure S2) [30, 31]. Therefore, high levels of methylation of *SLFN11* may explain the tendency for SLFN11 expression to be lower in older patients in our study.

### Translation of SLFN11 to the clinical practice

Our data showed that the prognosis of stage I (cT1b) patients was relatively good irrespective of SLFN11 status. In the USA, clinical stage IVA and IVB (cM1-lym) are indications for palliative therapy, and dCRT is not provided as a treatment option. In contrast, clinical stage II and III are generally indications for surgery or dCRT (as a non-surgical option). Importantly, the prognosis of patients treated with surgery is comparable to that of patients treated with dCRT [11, 33]. In our study setting, we showed that the SLFN11-dependent good outcome is more evident in clinical stage II and III patients than in those at other stages. Therefore, examination of SLFN11 expression level may be particularly useful for clinical stage II–III ESCC patients who wish to choose dCRT (instead of surgery) to preserve esophageal function. Moreover, this subset of patients is also a candidate for trimodal treatment, namely neoadjuvant concurrent chemoradiotherapy followed by surgery.

Taken together, our results suggest that the examination of SLFN11 expression might be useful in optimal treatment selection.

### Limitations

Our data should be interpreted in the context of several limitations.

First, because our study was retrospective, we could not address all confounding biases. In an attempt to minimize the influence of confounding biases, we recruited as many patients as possible (*n* = 597) and adopted a solid outcome endpoint (i.e. overall survival).

Second, from among these 597 patients, 73 were enrolled in accordance with the inclusion/exclusion criteria. Even in a study of this sample size, SLFN11 expression level was an independent prognostic factor, probably because of its strong correlation with DNA-damaging treatment sensitivity. Similar future studies should be conducted to confirm our conclusions.

Third, we provisionally defined an H-score ≥ 51 as indicating high SLFN11 expression.

This threshold was selected from an ROC curve of SLFN11 expression level versus 3-year overall survival after dCRT (data not shown). Therefore, the threshold may differ according to different settings such as the antibody used for immunohistochemistry, specimen size, and/or cancer type.

Fourth, we have used a low-dose nedaplatin + 5-fluorouracil regimen in dCRT for renal protection since 2003. Nedaplatin is used as an alternative to cisplatin, especially in Japan, as it reportedly has fewer side-effects than cisplatin [34]. According to national data from 2016 (Japanese Ministry of Health, Labor and Welfare), nedaplatin (instead of cisplatin) was used at a frequency of 12% (at 1 to 7.4 ratio) in chemo−/chemoradiotherapy in esophageal cancer patients [35]. Although cisplatin + 5-fluorouracil is the global standard combination regimen for dCRT in ESCC patients, the treatment performance of cisplatin + 5-fluorouracil with concomitant radiotherapy and low-dose nedaplatin + 5-fluorouracil with concomitant radiotherapy is reportedly similar in esophageal cancer patients [6, 34]. In this setting, as shown above, cancer cell lines with high SLFN11 expression had better therapeutic sensitivity to nedaplatin than those with low SLFN11 expression (Figure S1). Given that our aim was to investigate the effect of SLFN11 on the reactivity of ESCC to a platinum derivative and radiation, we do not think that enrolling patients treated with a low-dose nedaplatin + 5-fluorouracil regimen with radiation affected our conclusion.

Fifth, among the types of esophageal cancer, adenocarcinoma is the most prevalent in western countries, while ESCC is the most frequent (90.5%) in Japan [36]. Therefore, the frequency of different pathological types of esophageal cancer may differ according to ethnicity.

Sixth, although, SLFN11 expression was an independent prognostic factor in our own study cohort, external validation of this finding is warranted. Confirmation in a larger study may allow the drawing of firm conclusions.

Finally, in our vitro study, we investigated the association between SLFN11 expression and sensitivity to these anticancer agents using leukemia cell lines, as in our previous report, to allow timely reporting [18]. Additional evaluation in esophageal cancer cell lines is desirable.

## Conclusion

Our study revealed that high SLFN11 expression is associated with better prognosis in E-SCC patients treated with dCRT because SLFN11 sensitizes ESCC cells to DNA-damaging treatments such as platinum derivatives and radiation. We believe that examination of SLFN11 expression is useful in ensuring the optimal selection of anti-cancer treatment. The clinical predictive value of SLFN11 warrants further evaluation in prospective clinical studies.

## Supplementary Information

**Additional file 1: Table S1**. Key inclusion and exclusion criteria. **Figure S1**. SLFN11 sensitizes cancer cells to nedaplatin and carboplatin but not 5-fluorouracil. (A–C) Viability curves of the indicated cell lines after continuous treatment for 72 h with the indicated agents (carboplatin, nedaplatin, and 5-fluorouracil). ATP concentration was measured to estimate cell viability. The viability of untreated cells was defined as 100%. Error bars represent standard deviation (*n* = 3). Human leukemia CCRF-CEM SLFN11-proficient [parent] and -deficient [SLFN11-KO], and K562 SLFN11-deficient [K562 + vector] and -proficient [K562 + SLFN11] cell lines were established previously [18, 20, 31]. **Figure S2**. Inverse correlation between DNA methylation and transcripts of *SLFN11*. Scatter plot shows the level of *SLFN11* methylation in the promoter region (y-axis) and its mRNA expression level (Log2, x-axis) in esophageal squamous cell carcinoma cell lines within the dataset Sanger/MGH GDSC (http://discover.nci.nih.gov/cellminercdb). Pearson’s correlation coefficient (r) and two-sided *P* value (p) are shown above the chart. (PPTX 326 kb)

## Data Availability

The data that support the findings of this study are available from Hamamatsu University School of Medicine but restrictions apply to their availability: they were used under license for the present study, and so are not publicly available. Data are, however, available from the authors upon reasonable request and with the permission of Hamamatsu University School of Medicine.

## Abbreviations

Ce: Cervical esophagus
cisplatin: Cis-diamminedichloro-platinum (cisplatin)
CI: Confidence interval
cM1-lym: cM1 with distant lymph node metastasis
cN0: no regional lymph node metastasis
cN1: metastasis in 1–2 regional lymph nodes
cN2: metastasis in 3–6 regional lymph nodes
cN3: metastasis in 7 or more regional lymph nodes
CR: Complete response
cT1a: Tumor invades the muscularis mucosa
cT1b: Tumor invades the submucosa
cT2: Tumor invades the muscularis propria
cT3: Tumor invades the adventitia
cT4a: Tumor invades the pleura, pericardium, azygos vein, diaphragm, or peritoneum
cT4b: Tumor invades other adjacent structures, such as the aorta, vertebral body, or trachea
dCRT: Definitive chemoradiotherapy
ECOG: Eastern Cooperative Oncology Group
eGFR: Estimated glomerular filtration rate
EGJ: Esophago-gastric junction
ESCC: Esophageal squamous cell carcinoma
H-score: histo-score
HR: Hazard ratio
Lt: Lower thoracic esophagus
Mt: Middle thoracic esophagus
N/A: Not applicable
PR: Partial response
RECIST: Response Evaluation Criteria in Solid Tumors
SCC: Squamous cell carcinoma
SD: Stable disease
SLFN-11: Schlafen-11
UICC: Union for International Cancer Control
Ut: Upper thoracic esophagus

## References

[1] Herskovic A, Martz K, al-Sarraf M, Leichman L, Brindle J, Vaitkevicius V (1992). Combined chemotherapy and radiotherapy compared with radiotherapy alone in patients with cancer of the esophagus. N Engl J Med.

[2] Cooper JS, Guo MD, Herskovic A, Macdonald JS, Martenson JA, Al-Sarraf M (1999). Chemoradiotherapy of locally advanced esophageal cancer: long-term follow-up of a prospective randomized trial (RTOG 85-01). Radiation Therapy Oncology Group. Jama.

[3] Ishida K, Ando N, Yamamoto S, Ide H, Shinoda M (2004). Phase II study of cisplatin and 5-fluorouracil with concurrent radiotherapy in advanced squamous cell carcinoma of the esophagus: a Japan esophageal oncology group (JEOG)/Japan clinical oncology group trial (JCOG9516). Jpn J Clin Oncol.

[4] Ohtsu A, Boku N, Muro K, Chin K, Muto M, Yoshida S (1999). Definitive chemoradiotherapy for T4 and/or M1 lymph node squamous cell carcinoma of the esophagus. J Clin Oncol..

[5] Nishimura Y, Suzuki M, Nakamatsu K, Kanamori S, Yagyu Y, Shigeoka H (2002). Prospective trial of concurrent chemoradiotherapy with protracted infusion of 5-fluorouracil and cisplatin for T4 esophageal cancer with or without fistula. Int J Radiat Oncol Biol Phys.

[6] Shinoda M, Ando N, Kato H, Tsubosa Y, Minashi K, Watanabe G (2010). A multicenter randomized phase II (rPII)/III study comparing concurrent chemoradiotherapy (CRT) with low-dose cisplatin plus continuous infusion of 5-fluorouracil (LDPF) and standard-dose PF (SDPF) for locally advanced unresectable squamous cell carcinoma of the thoracic esophagus (JCOG0303). J Clin Oncol.

[7] Kitagawa Y, Uno T, Oyama T, Kato K, Kato H, Kawakubo H, et al. Esophageal cancer practice guidelines 2017 edited by the Japan Esophageal Society: part 1. Esophagus : official journal of the Japan Esophageal Society. 2019;16(1):1-24. 10.1007/s10388-018-0641-9 PMC6510883 30171413

[8] Nishimura Y, Mitsumori M, Hiraoka M, Koike R, Nakamatsu K, Kawamura M (2009). A randomized phase II study of cisplatin/5-FU concurrent chemoradiotherapy for esophageal cancer: short-term infusion versus protracted infusion chemotherapy (KROSG0101/JROSG021). Radiother Oncol.

[9] Nishimura Y, Hiraoka M, Koike R, Nakamatsu K, Itasaka S, Kawamura M, et al. Long-term follow-up of a randomized Phase II study of cisplatin/5-FU concurrent chemoradiotherapy for esophageal cancer (KROSG0101/JROSG021). Jpn J Clin Oncol. 2012;42(9):807-12. 10.1093/jjco/hys112 22811410

[10] Kato H, Sato A, Fukuda H, Kagami Y, Udagawa H, Togo A (2009). A phase II trial of chemoradiotherapy for stage I esophageal squamous cell carcinoma: Japan clinical oncology group study (JCOG9708). Jpn J Clin Oncol.

[11] Kato K, Muro K, Minashi K, Ohtsu A, Ishikura S, Boku N (2011). Phase II study of chemoradiotherapy with 5-fluorouracil and cisplatin for stage II-III esophageal squamous cell carcinoma: JCOG trial (JCOG 9906). Int J Radiat Oncol Biol Phys.

[12] Kato K, Nakajima TE, Ito Y, Katada C, Ishiyama H, Tokunaga SY (2013). Phase II study of concurrent chemoradiotherapy at the dose of 50.4 Gy with elective nodal irradiation for stage II-III esophageal carcinoma. Jpn J Clin Oncol.

[13] Murai J, Thomas A, Miettinen M, Pommier Y. Schlafen 11 (SLFN11), a restriction factor for replicative stress induced by DNA-targeting anti-cancer therapies. Pharmacol Ther. 2019;201:94-102. 10.1016/j.pharmthera.2019.05.009 PMC6708787 31128155

[14] Mavrommatis E, Fish EN, Platanias LC (2013). The schlafen family of proteins and their regulation by interferons. J Interferon Cytokine Res.

[15] Zoppoli G, Regairaz M, Leo E, Reinhold WC, Varma S, Ballestrero A (2012). Putative DNA/RNA helicase Schlafen-11 (SLFN11) sensitizes cancer cells to DNA-damaging agents. Proc Natl Acad Sci U S A.

[16] Barretina J, Caponigro G, Stransky N, Venkatesan K, Margolin AA, Kim S (2012). The Cancer cell line encyclopedia enables predictive modelling of anticancer drug sensitivity. Nature..

[17] Lok BH, Gardner EE, Schneeberger VE, Ni A, Desmeules P, Rekhtman N (2017). PARP inhibitor activity correlates with SLFN11 expression and demonstrates synergy with Temozolomide in small cell lung Cancer. Clin Cancer Res..

[18] Murai J, Feng Y, Yu GK, Ru Y, Tang SW, Shen Y (2016). Resistance to PARP inhibitors by SLFN11 inactivation can be overcome by ATR inhibition. Oncotarget..

[19] Allison Stewart C, Tong P, Cardnell RJ, Sen T, Li L, Gay CM (2017). Dynamic variations in epithelial-to-mesenchymal transition (EMT), ATM, and SLFN11 govern response to PARP inhibitors and cisplatin in small cell lung cancer. Oncotarget..

[20] Murai J, Tang SW, Leo E, Baechler SA, Redon CE, Zhang H (2018). SLFN11 Blocks Stressed Replication Forks Independently of ATR. Molecular Cell.

[21] Mu Y, Lou J, Srivastava M, Zhao B, Feng XH, Liu T (2016). SLFN11 inhibits checkpoint maintenance and homologous recombination repair. EMBO Rep.

[22] McShane LM, Altman DG, Sauerbrei W, Taube SE, Gion M, Clark GM (2005). REporting recommendations for tumour MARKer prognostic studies (REMARK). Eur J Cancer.

[23] Pietanza MC, Waqar SN, Krug LM, Dowlati A, Hann CL, Chiappori A (2018). Randomized, double-blind, phase II study of Temozolomide in combination with either Veliparib or placebo in patients with relapsed-sensitive or refractory small-cell lung Cancer. J Clin Oncol.

[24] Kim SH, Park WS, Park EY, Park B, Joo J, Joung JY (2017). The prognostic value of BAP1, PBRM1, pS6, PTEN, TGase2, PD-L1, CA9, PSMA, and Ki-67 tissue markers in localized renal cell carcinoma: a retrospective study of tissue microarrays using immunohistochemistry. PLoS One.

[25] Choueiri TK, Figueroa DJ, Fay AP, Signoretti S, Liu Y, Gagnon R (2015). Correlation of PD-L1 tumor expression and treatment outcomes in patients with renal cell carcinoma receiving sunitinib or pazopanib: results from COMPARZ, a randomized controlled trial. Clin Cancer Res..

[26] Oken MM, Creech RH, Tormey DC, Horton J, Davis TE, McFadden ET (1982). Toxicity and response criteria of the eastern cooperative oncology group. Am J Clin Oncol.

[27] Kagami T, Yamade M, Suzuki T, Uotani T, Tani S, Hamaya Y (2018). High expression level of CD44v8-10 in cancer stem-like cells is associated with poor prognosis in esophageal squamous cell carcinoma patients treated with chemoradiotherapy. Oncotarget..

[28] Minsky BD, Pajak TF, Ginsberg RJ, Pisansky TM, Martenson J, Komaki R (2002). INT 0123 (radiation therapy oncology group 94-05) phase III trial of combined-modality therapy for esophageal cancer: high-dose versus standard-dose radiation therapy. J Clin Oncol.

[29] Ariga H, Nemoto K, Miyazaki S, Yoshioka T, Ogawa Y, Sakayauchi T (2009). Prospective comparison of surgery alone and chemoradiotherapy with selective surgery in resectable squamous cell carcinoma of the esophagus. Int J Radiat Oncol Biol Phys.

[30] Nogales V, Reinhold WC, Varma S, Martinez-Cardus A, Moutinho C, Moran S (2016). Epigenetic inactivation of the putative DNA/RNA helicase SLFN11 in human cancer confers resistance to platinum drugs. Oncotarget..

[31] Tang SW, Thomas A, Murai J, Trepel JB, Bates SE, Rajapakse VN (2018). Overcoming resistance to DNA-targeted agents by epigenetic activation of Schlafen 11 (SLFN11) expression with class I histone Deacetylase inhibitors. Clin Cancer Res.

[32] He T, Zhang M, Zheng R, Zheng S, Linghu E, Herman JG (2017). Methylation of SLFN11 is a marker of poor prognosis and cisplatin resistance in colorectal cancer. Epigenomics..

[33] Stahl M, Stuschke M, Lehmann N, Meyer HJ, Walz MK, Seeber S (2005). Chemoradiation with and without surgery in patients with locally advanced squamous cell carcinoma of the esophagus. J Clin Oncol.

[34] Saito H, Ohta A, Abe E, Kaidu M, Shioi M, Nakano T (2017). Definitive chemoradiotherapy with low-dose continuous 5-fluorouracil reduces hematological toxicity without compromising survival in esophageal squamous cell carcinoma patients. Clin Transl Radiat Oncol.

[35] Medical Economics Division HIB, Japanese Ministry of Health Labor and Welfare. Annual report 2016 on discharged patients survey (in Japanese). Available at: https://www.mhlwgojp/file/05-Shingikai-12404000-Hokenkyoku-Iryouka/0000196184.pdf. Accessed 1 May 2019.

[36] Tachimori Y, Ozawa S, Numasaki H, Fujishiro M, Matsubara H, Oyama T (2016). Comprehensive registry of esophageal Cancer in Japan, 2009. Esophagus.

