# Supplementary material for: The first evidence for SLFN11 expression as an independent prognostic factor for patients with esophageal cancer after chemoradiotherapy
Source: BMC Cancer. 2020 Nov 20;20:1123. doi: 10.1186/s12885-020-07574-x (PMC7678160; doi:10.1186/s12885-020-07574-x)
Supplement: Supplementary file 1 — Additional file 1: Table S1. Key inclusion and exclusion criteria. Figure S1. SLFN11 sensitizes cancer cells to nedaplatin and carboplatin but not 5-fluorouracil. (A–C) Viability curves of the indicated cell lines after continuous treatment for 72 h with the indicated agents (carboplatin, nedaplatin, and 5-fluorouracil). ATP concentration was measured to estimate cell viability. The viability of untreated cells was defined as 100%. Error bars represent standard deviation (n = 3). Human leukemia CCRF-CEM SLFN11-proficient [parent] and -deficient [SLFN11-KO], and K562 SLFN11-deficient [K562 + vector] and -proficient [K562 + SLFN11] cell lines were established previously [18, 20, 31]. Figure S2. Inverse correlation between DNA methylation and transcripts of SLFN11. Scatter plot shows the level of SLFN11 methylation in the promoter region (y-axis) and its mRNA expression level (Log2, x-axis) in esophageal squamous cell carcinoma cell lines within the dataset Sanger/MGH GDSC (http://discover.nci.nih.gov/cellminercdb). Pearson’s correlation coefficient (r) and two-sided P value (p) are shown above the chart. (PPTX 326 kb) [file 12885_2020_7574_MOESM1_ESM.pptx]

## Slide 1
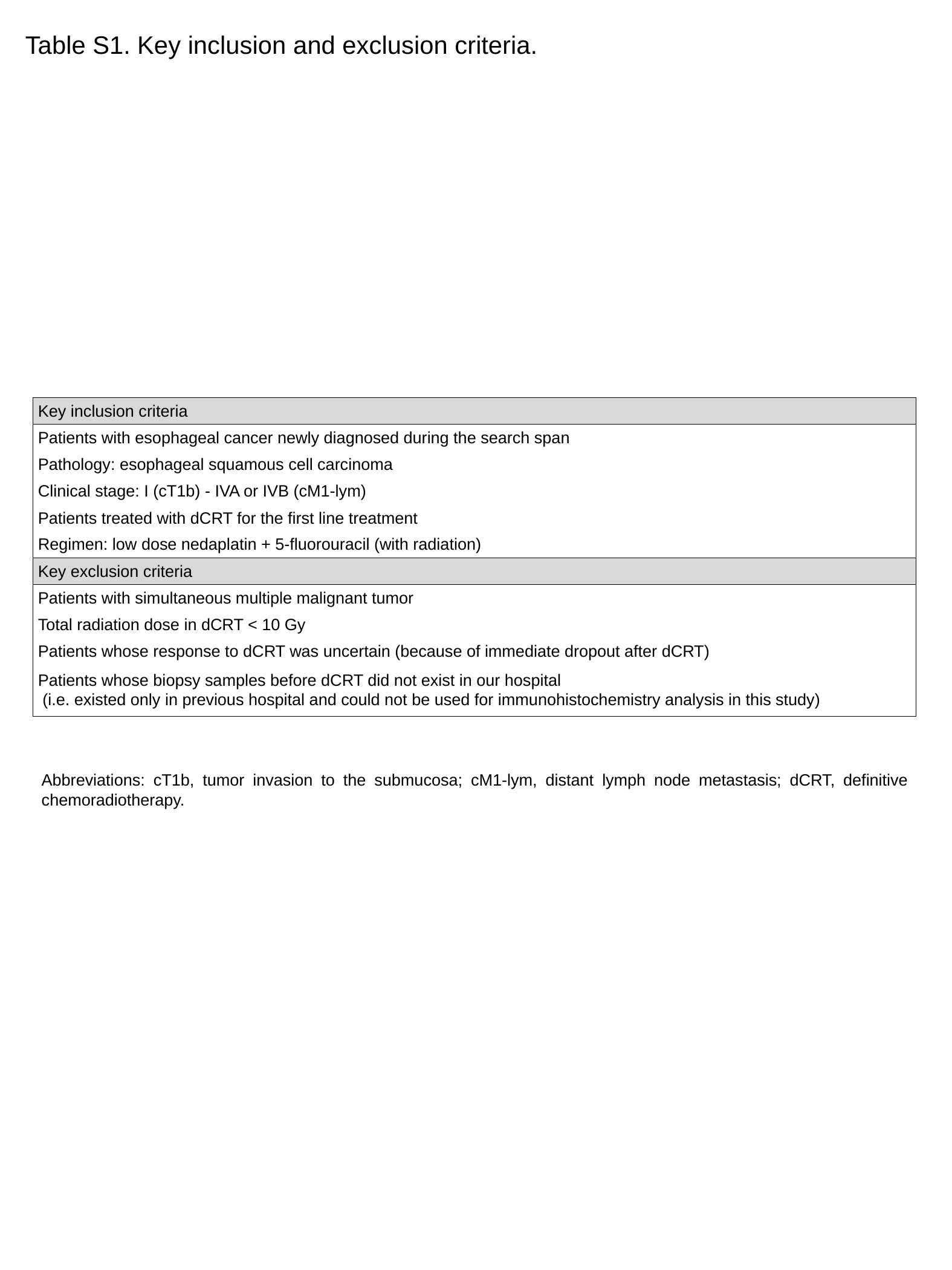

Table S1. Key inclusion and exclusion criteria.
| Key inclusion criteria |
| --- |
| Patients with esophageal cancer newly diagnosed during the search span |
| Pathology: esophageal squamous cell carcinoma |
| Clinical stage: I (cT1b) - IVA or IVB (cM1-lym) |
| Patients treated with dCRT for the first line treatment |
| Regimen: low dose nedaplatin + 5-fluorouracil (with radiation) |
| Key exclusion criteria |
| Patients with simultaneous multiple malignant tumor |
| Total radiation dose in dCRT < 10 Gy |
| Patients whose response to dCRT was uncertain (because of immediate dropout after dCRT) |
| Patients whose biopsy samples before dCRT did not exist in our hospital |
| (i.e. existed only in previous hospital and could not be used for immunohistochemistry analysis in this study) |
Abbreviations: cT1b, tumor invasion to the submucosa; cM1-lym, distant lymph node metastasis; dCRT, definitive chemoradiotherapy.

## Slide 2
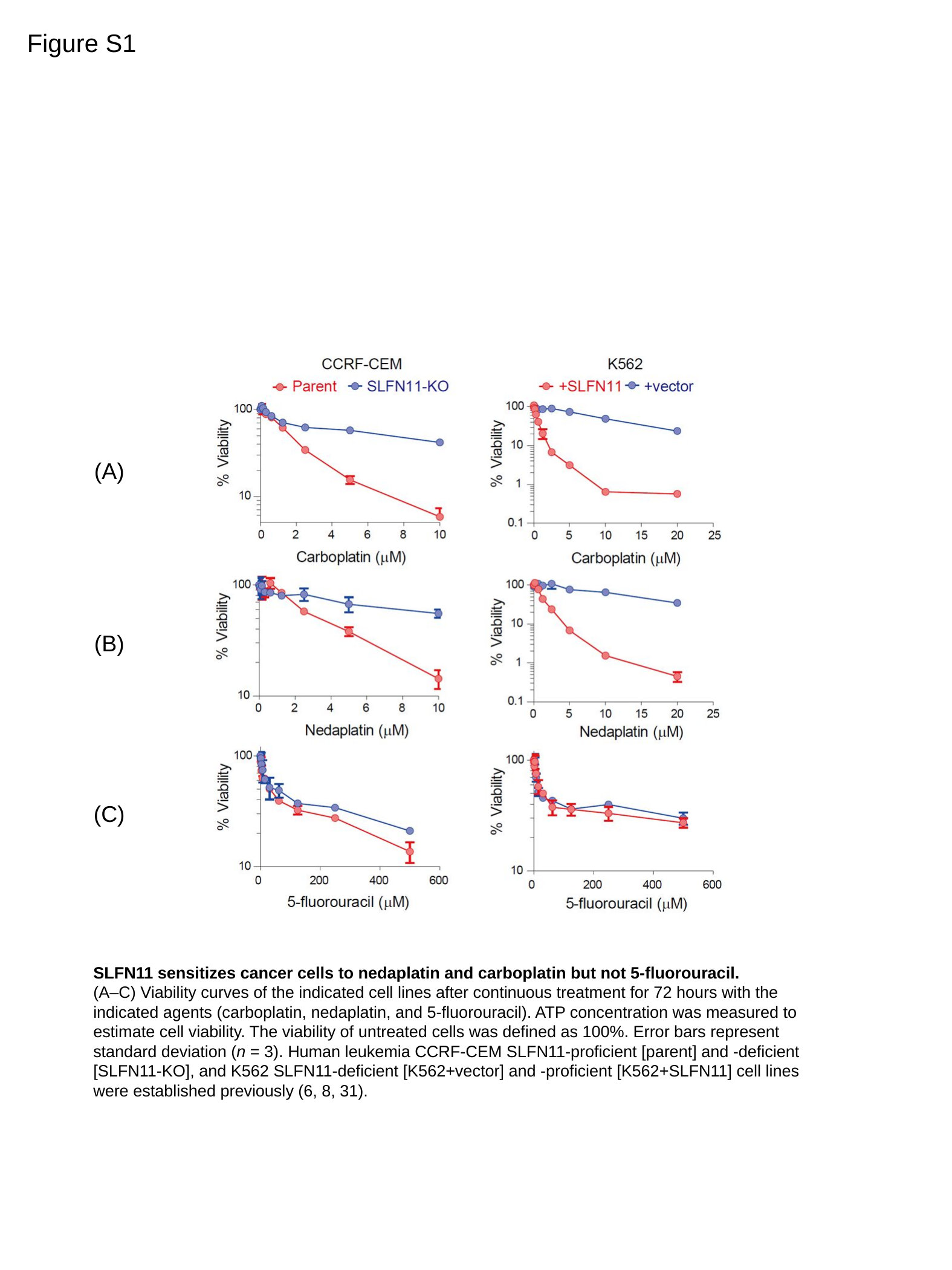

Figure S1
(A)
(B)
(C)
SLFN11 sensitizes cancer cells to nedaplatin and carboplatin but not 5-fluorouracil.
(A–C) Viability curves of the indicated cell lines after continuous treatment for 72 hours with the indicated agents (carboplatin, nedaplatin, and 5-fluorouracil). ATP concentration was measured to estimate cell viability. The viability of untreated cells was defined as 100%. Error bars represent standard deviation (n = 3). Human leukemia CCRF-CEM SLFN11-proficient [parent] and -deficient [SLFN11-KO], and K562 SLFN11-deficient [K562+vector] and -proficient [K562+SLFN11] cell lines were established previously (6, 8, 31).

## Slide 3
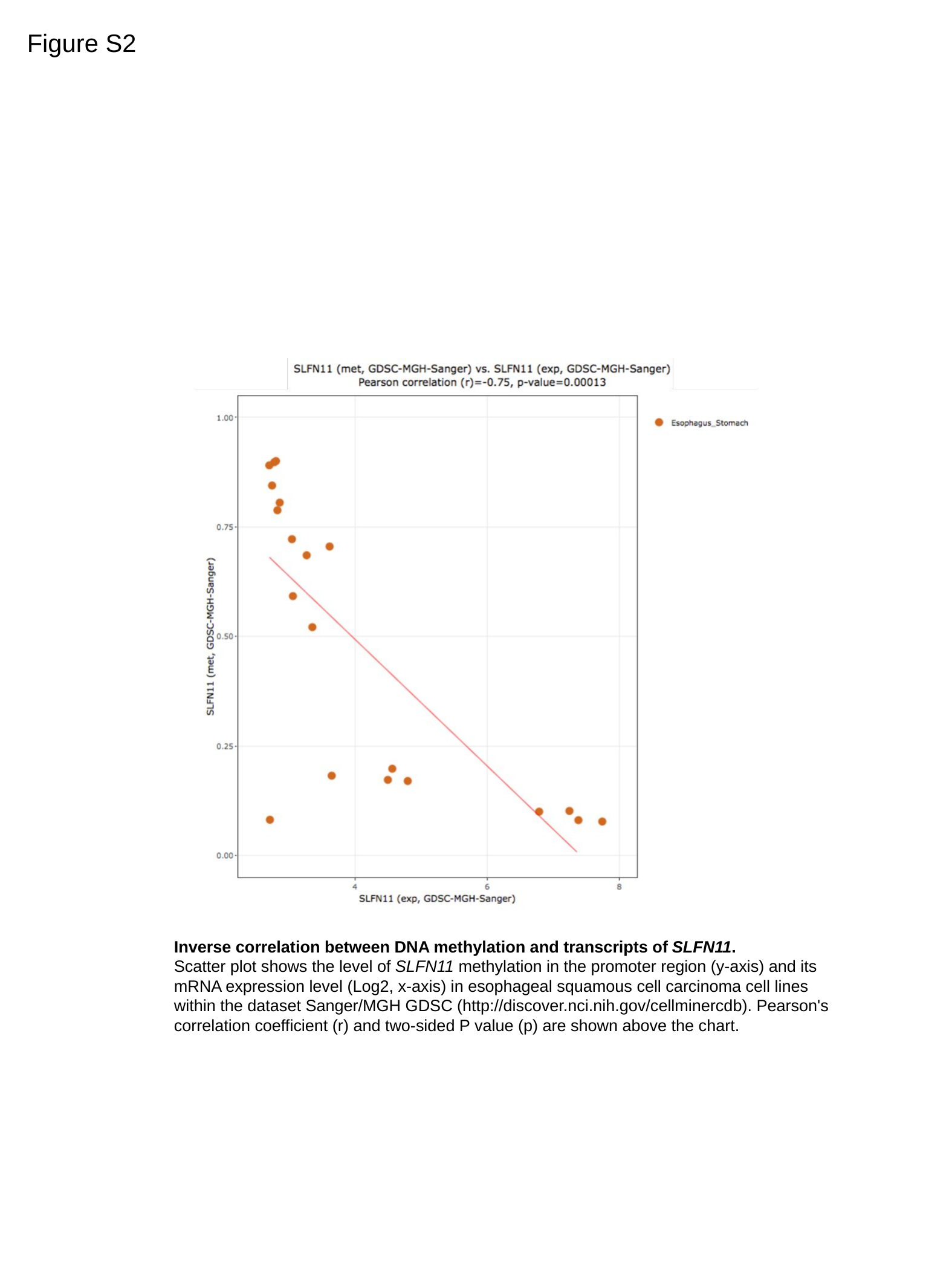

Figure S2
Inverse correlation between DNA methylation and transcripts of SLFN11.
Scatter plot shows the level of SLFN11 methylation in the promoter region (y-axis) and its mRNA expression level (Log2, x-axis) in esophageal squamous cell carcinoma cell lines within the dataset Sanger/MGH GDSC (http://discover.nci.nih.gov/cellminercdb). Pearson's correlation coefficient (r) and two-sided P value (p) are shown above the chart.
